# Inferring Personality From Social Media Activity Using Large Language Models: Cross‐Model Agreement, Temporal Stability, and Convergent Validity With Self‐Reports

**DOI:** 10.1111/jopy.70019

**Published:** 2025-09-02

**Authors:** Davide Marengo, Christian Montag, Michele Settanni

**Affiliations:** ^1^ Department of Psychology University of Turin Turin Italy; ^2^ Centre for Cognitive and Brain Sciences, Institute of Collaborative Innovation University of Macau Macau SAR China; ^3^ Department of Computer and Information Science, Faculty of Science and Technology University of Macau Macau SAR China; ^4^ Department of Psychology, Faculty of Social Sciences University of Macau Macau SAR China

**Keywords:** big five personality traits, computational social science, digital footprints, large language models, psychoinformatics

## Abstract

**Introduction:**

Large language models (LLMs) offer a promising approach to infer personality traits unobtrusively from digital footprints. However, the reliability and validity of these inferences remain underexplored.

**Method:**

Gemini 1.5 Pro and GPT‐4o were used to infer Big Five traits from 2 years of Facebook posts by 1214 Italian users. Predictions were compared to self‐reports on the Ten‐Item Personality Inventory.

**Results:**

LLM predictions underestimated Agreeableness and Conscientiousness, overestimated Extraversion, while Neuroticism and Openness closely aligned with self‐report means. On repeated prompting, Gemini 1.5 Pro inferences showed less variability than GPT‐4o, with both models achieving excellent reliability when aggregating inferences. Temporal stability was highest when combining predictions across LLMs, with test–retest correlations over 2 years ranging from 0.44 for Conscientiousness to 0.60 for Openness. Cross‐LLM agreement was highest when combining inferences from multiple time points, with correlations ranging from 0.58 for Neuroticism to 0.83 for Extraversion. Correlations with self‐reports were modest, reaching 0.27 for Extraversion, 0.24 for Agreeableness, 0.23 for Conscientiousness, 0.18 for Neuroticism, and 0.31 for Openness when combining LLM inferences across LLMs and time points.

**Conclusion:**

These findings advance understanding of LLMs' potential for personality inference, highlighting the importance of aggregating inferences to enhance the reliability and validity of such assessments.

## 1 Introduction

Predicting psychological traits from digital footprints has long been a goal of computational social science and the field of *Psychoinformatics* (Montag et al. 2016). Early studies demonstrated that supervised machine learning models could extract personality information from online behaviors such as Facebook Likes, status updates, and browsing histories (Golbeck et al. 2011; Qiu et al. 2012). These studies often achieved promising correlations with self‐reported personality traits, such as the Big Five dimensions, through the application of algorithms trained on large datasets of user behavior paired with psychometric assessments (Kosinski et al. 2013; Azucar et al. 2018). For example, Park et al. (2015) used social media language to develop automated personality assessments, achieving levels of accuracy that approached or surpassed human predictions based on limited cues.

Despite their success, these earlier approaches were constrained by their reliance on pre‐defined feature extraction and training data. The advent of large language models (LLMs) such as OpenAI's GPT series and Gemini 1.5 Pro marked a significant leap forward. Unlike traditional models, LLMs leverage extensive pre‐training on diverse text corpora, enabling them to perform zero‐shot and few‐shot inference without requiring task‐specific training data (Brown et al. 2020; Radford et al. 2019). The potential of LLMs for applications in mental health has recently gained attention (Malgaroli et al. 2025). As noted by Volkmer et al. (2024), LLMs offer new avenues for scalable, unobtrusive monitoring of psychological states and problematic behaviors, which could improve early identification of risk factors and screening by leveraging data sources such as social media activity. Recent studies combining LLMs with social media data have yielded promising results, demonstrating strong convergence between LLM‐based inferences and human evaluations in mental health triaging (e.g., Settanni et al. 2025), as well as between LLM inferences and self‐reported behaviors (e.g., Marengo et al. 2025). In personality research, Peters and Matz (2024) exemplify this shift by showing that LLMs can infer Big Five personality traits directly from Facebook status updates, achieving correlations comparable to earlier supervised models. However, it is important to note that Peters and Matz did not examine key aspects such as the stability of inferences across repeated LLM calls, their temporal consistency when assessed using consecutive user data, or the agreement between different LLMs in predicting personality traits. Addressing these gaps is crucial for understanding the robustness and reliability of using LLMs for personality assessment.

In light of these considerations, the primary objectives of the present study are to determine the feasibility of inferring Big Five traits from social media text using LLMs, and to evaluate the stability of these inferences across repeated prompting, over time, and across distinct models. Given the limited prior research in this area, our work is conducted with an exploratory approach. By doing so, we aim to provide a more comprehensive understanding of the reliability and potential limitations of applying LLMs to infer personality from naturalistic online behavior. To this end, we compare the ability of two leading LLMs, namely OpenAI's GPT‐4o and Google's Gemini 1.5 Pro, to infer Big Five personality traits based on individuals' Facebook activity spanning two consecutive years. The actual personality traits were measured using the Ten‐Item Personality Inventory (TIPI), a self‐report questionnaire completed once by participants. Importantly, the Facebook data analyzed covered the 2 years preceding participants' completion of the personality test, allowing us to assess whether LLMs can accurately infer personality traits from past behavior and maintain consistency in these predictions over time. It is also worth noting that our data, collected in 2018, are considerably more recent than those used by Peters and Matz (2024), who relied on the MyPersonality dataset, which included data collected between 2007 and 2012. Through this investigation, we aim to shed light on the reliability and validity of LLMs for unobtrusive personality assessment and provide insights into the implications for their use in research, industry applications, and ethical considerations (Kosinski 2024; Matz et al. 2020).

## 2 Materials & Methods

### 2.1 Participants and Procedure

Participants were recruited via a snowball sampling method among Italian Facebook users using a custom Facebook application. Enrollment required participants to be at least 18 years old and grant access to their Facebook data via the Facebook login. Informed consent was obtained from all participants. Data collection occurred between March and June 2018. For the present study, only participants for whom up to 24 months of posting activity were included. The sample consists of 1214 participants, with a majority of females (74.4%, or 903 participants) compared to males (25.6%, or 311 participants). In terms of age distribution, the largest group is composed of individuals aged 18–25 years, accounting for 73.6% of the sample (894 participants). Smaller proportions of the sample fall into older age groups: 18.6% are aged 26–30 years (226 participants), 3.6% are aged 31–35 years (44 participants), and 4.1% are aged 36 or older (50 participants). Regarding educational attainment, 52.1% of participants (631 individuals) have a high school diploma, 31.4% (381 participants) hold a bachelor's degree, and 13.9% (169 participants) have a master's degree. A small portion of the sample, 2.7% (33 participants), had only completed middle school. This distribution indicates a predominance of young, relatively well‐educated individuals, with the largest groups holding at least a high school diploma or bachelor's degree.

Facebook posting activity was gathered through the Facebook Graph API, which was at this time still open for the present research question. Data covered the 24 months preceding each participant's app access and questionnaire completion. In the 12 months preceding questionnaire completion (*T*_0_), participants posted an average of 35.06 Facebook entries (SD = 30.45). Data from the preceding year (*T*_−1_) showed slightly higher average activity, with 59.28 entries (SD = 44.66). Each year's data comprises a variable number of Facebook entries, reflecting the individual's activity level on the platform. Each entry includes a timestamp indicating the date and time of activity, a status update field containing short text‐based posts, a story description field detailing any shared content or actions taken, and a count of received Likes for the entry. Of note, the present data have been analyzed in different contexts and with different research questions elsewhere (Marengo et al. 2020; Marengo, Azucar, et al. 2021; Marengo, Montag, et al. 2021, 2022; Marengo, Settanni, and Montag 2022; Marengo and Settanni 2024a, 2024b). Informed consent was obtained from all individual participants included in the study. The research received ethical approval (*n* = 88,721) from the institutional review board of the University of Turin, Italy.

### 2.2 Self‐Report Big Five Personality

Personality differences were assessed by administering the Italian adaptation (Chiorri et al. 2015) of the Ten‐Item Personality Inventory (TIPI) (Gosling et al. 2003), a short measure assessing the Big‐Five personality traits of Extraversion, Agreeableness, Conscientiousness, Neuroticism (as reverse Emotional Stability), Openness to new experiences. The instrument consists of 10 items (2 items per trait) with a common stem of ‘I see myself as’. Each item is rated on a 7‐point scale ranging from 1 (*disagree strongly*) to 7 (*agree strongly*). In spite of its brevity, the TIPI personality scales have shown good psychometric properties when compared to longer instruments assessing Big Five personality traits (Gosling et al. 2003). Given the low number of items per trait, internal consistency for this instrument is expected to be quite low and to deviate significantly across traits (and one could argue to not consider it at all, but rely more on external validity and so forth); in our study, based on Cronbach's alpha, internal consistency ranged from 0.32 to 0.70 (Extraversion: *α* = 0.70; Agreeableness: *α* = 0.32, Conscientiousness: *α* = 0.56; Neuroticism: *α* = 0.53; Openness: *α* = 0.42). However, the observed values are in line with the ones presented in TIPI validation studies (e.g., Gosling et al. 2003; Romero et al. 2012), including the Italian validation by Chiorri et al. (2015).

### 2.3 Personality Inferences From LMM

Personality inferences were obtained by submitting a specifically devised prompt to two large language models: Google's Gemini 1.5 Pro and OpenAI's GPT‐4o. Prompts were sent via the official APIs for both models, with the temperature set at 0.5 to balance coherence and variability. Importantly, no memory or conversation history was retained between prompts, ensuring that each inference was independent and based solely on the provided input. For each participant and each year of Facebook data (*T*_0_ and *T*_−1_), the same prompt structure was used, consisting of three main sections:

1. *Personality Trait Definitions*. The prompt began by providing descriptions of the five personality traits being assessed: Extraversion, Agreeableness, Conscientiousness, Neuroticism (reverse Emotional Stability), and Openness to Experience. Each trait was defined using illustrative adjectives describing high and low levels, drawing on facets and trait descriptors from the BFI‐44 (John et al. 1991). For instance, a person high in Extraversion was described as “talkative, sociable, energetic, or enthusiastic,” while someone low in Extraversion was described as “reserved, quiet, or inhibited.” Similar descriptions were provided for the other four traits, covering both high and low ends of each dimension. These definitions served as the foundation for the LLMs to interpret the Facebook data and make personality inferences. Importantly, this section of the prompt did not include examples of scored Facebook data or sample ratings, meaning the LLMs were not shown how to link text data to trait scores. This preserved a zero‐shot setup, requiring the models to infer personality solely based on the trait definitions and the participants' Facebook posts.
2. *Facebook Data*: The prompt then presented the participant's Facebook data for the relevant year. This data included a chronologically ordered list of entries, each with a timestamp, status update (text), story update (description of shared content), and the number of Likes received. The number of entries varied per participant.
3. *Rating Instructions*: The prompt concluded with instructions to rate the participant on each of the five personality traits using a 7‐point Likert scale, where 1 indicated “very low,” 2 “low,” 3 “low to moderate,” 4 “moderate,” 5 “moderate to high,” 6 “high,” and 7 “very high.” The models were explicitly instructed to provide their ratings as a comma‐separated list, without any additional explanations or justifications.

This standardized prompt structure was used consistently across all participants, years, and both LLMs to ensure uniformity in data presentation and response format. In following previous studies (e.g., Cao and Kosinski 2024; Peters and Matz 2024), each prompt was submitted in multiple *k* independent iterations per participant and timepoint to increase measurement precision and assess the reliability of predictions; in the present study, *k* = 10 prompt repetitions were performed for each condition. The full structure of the prompt is provided in Supporting Information.

### 2.4 Data Analysis Strategy

First, we examined the distribution of personality inferences generated by each LLM (Gemini 1.5 Pro and GPT‐4o) when prompted with participants' Facebook data from the same year (*T*_0_) or the previous year (*T*_−1_) relative to the self‐report assessment. In these preliminary analyses, for all combinations of LLM and submitted data, we analyzed the means and standard deviations of the k = 10 repeated LLM outputs to assess how predicted trait averages aligned with participants' self‐reported trait means and to quantify the variability of predictions across repetitions. To further examine the stability of LLM inferences across repetitions, and in line with previous studies evaluating repeated LLM outputs (e.g., Bodroža et al. 2024; Schoenegger et al. 2025), we assessed the reliability of predictions by calculating intraclass correlation coefficients (ICCs). For each model and time point, we used a two‐way random‐effects model with absolute agreement, under the assumption that the multiple LLM iterations represented a random sample of possible outputs (Shrout and Fleiss 1979). We computed ICC(2,1) to estimate the reliability of single inferences across the *k* = 10 repetitions and ICC(2,*k*) to estimate the reliability of averaged predictions. This step allowed us to quantify the inherent variability of each LLM's outputs and the potential gains in precision achieved by averaging repeated prompts. Based on these preliminary analyses, all subsequent analyses were conducted using the averages of the 10 repetitions for each participant and trait to ensure greater measurement reliability (see Section 3.1).

Second, we examined the temporal stability of LLM‐derived personality inferences by calculating Spearman correlations (ρ) between average predictions from *T*_0_ and *T*_−1_ for each LLM. Additionally, we investigated the correlations across years of the average personality inferences obtained by combining the two models. Third, cross‐model agreement was assessed by correlating personality inferences generated by Gemini 1.5 Pro and GPT‐4o at both *T*_0_ and *T*_−1_, as well as by examining agreement using average personality inferences across both years for each model.

Fourth, we evaluated the validity of LLM inferences by correlating them with self‐reported personality measures from the TIPI. This analysis was conducted separately for each LLM, for each year of Facebook data (i.e., *T*_0_ and *T*_−1_), and for combined inferences across years and models. Both raw and disattenuated correlations were reported to account for measurement error in the TIPI. All correlation analyses controlled for sex and age, which were included as covariates to partial out their potential influence on associations involving personality inferences.

Finally, we evaluated the accuracy of LLM‐derived predictions of Big Five traits by computing mean absolute error (MAE), mean squared error (MSE), and root mean squared error (RMSE) against participants' self‐reported scores. Analyses were conducted separately for each LLM predictor, including Gemini 1.5 pro and GPT4o at *T*_0_ and *T*_−1_, and their combined averages. To establish a chance‐level benchmark, we generated random predictions uniformly sampled from the self‐report scale range (1–7) for each participant and trait, repeating this 10 times to obtain distributions of baseline errors. For each random repetition, predictive *R* ^2^ was calculated as the proportion of variance explained by the LLM predictions relative to random guesses. All analyses were conducted in Python 3.10 using *pandas* and *numpy*. We summarized predictive R^2^ for each LLM by reporting the mean, minimum, and maximum across the 10 repetitions.

## 3 Results

### 3.1 Distribution of Personality Inferences and Stability Across Repeated Iterations

**FIGURE 1:**
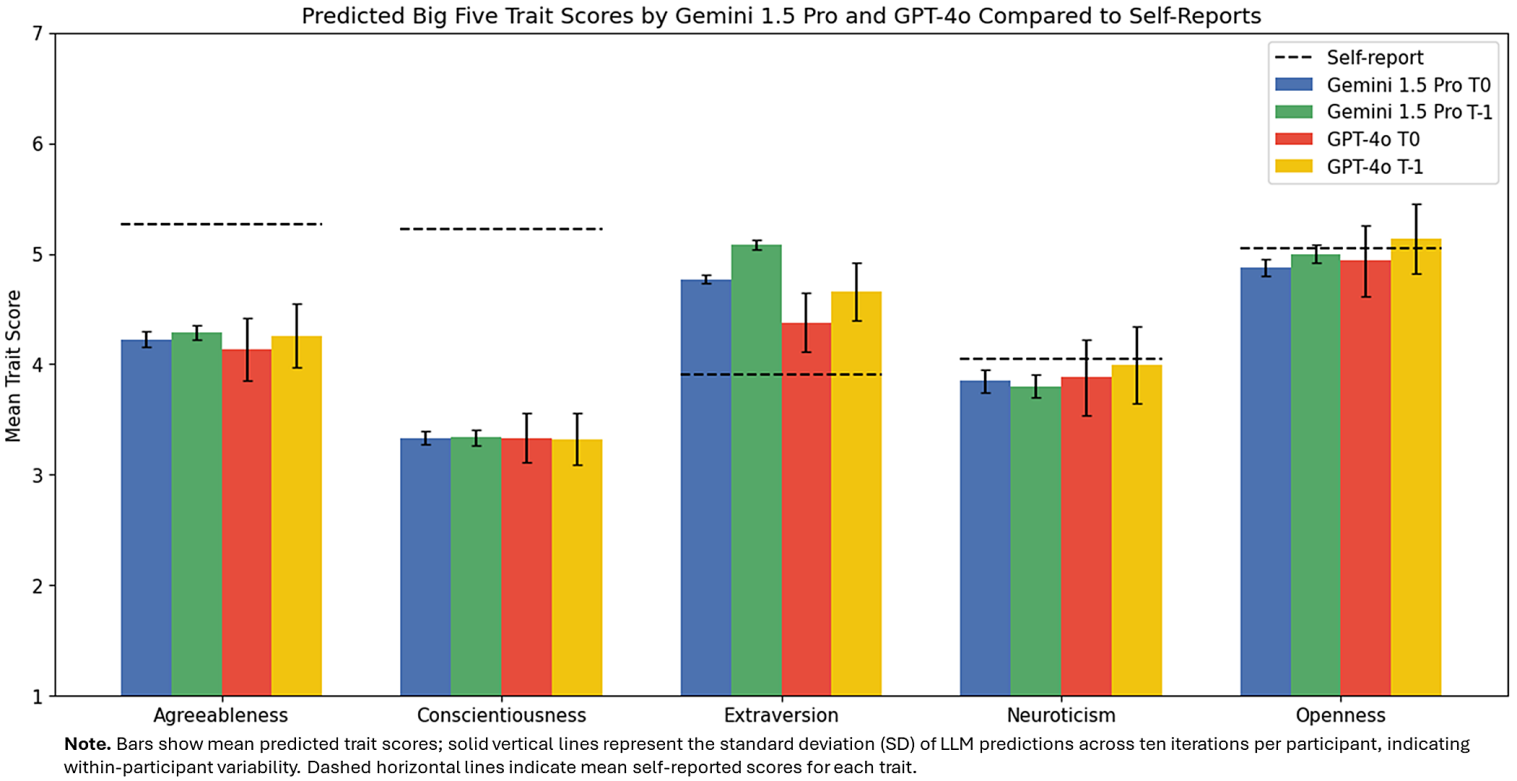
Predicted Big Five trait scores by Gemini 1.5 Pro and GPT‐4o compared to self‐reports.

First, we examined the distribution of the personality inferences obtained by querying the LLMs across 10 repeated iterations. The bar chart in Figure 1 visualizes the distribution of these inferences and how predicted trait scores compare to self‐reported means (shown as dashed horizontal lines), with vertical solid lines representing the standard deviation (SD) of LLM predictions across k = 10 repetitions per participant. Notably, Agreeableness and Conscientiousness were consistently underestimated by both Gemini 1.5 Pro and GPT‐4o at both time points, with predicted means substantially lower than self‐reports. In contrast, Extraversion was generally overestimated across models, especially by Gemini 1.5 Pro, although the overestimation was less pronounced than the underestimation observed for Agreeableness and Conscientiousness. In turn, predictions for Neuroticism and Openness were more closely aligned with self‐reports, with GPT‐4o inferences nearly matching self‐reported means for both traits. Notably, the solid vertical lines highlight that for all traits, GPT‐4o produced less stable predictions across repetitions compared to the more consistent estimates generated by Gemini 1.5 Pro.

To quantify further the stability of LLM‐derived personality inferences, we calculated intraclass correlation coefficients (ICCs) using a two‐way random‐effects model with absolute agreement (ICC[2,1] for single iterations and ICC[2,*k*] for averages). These analyses assessed the consistency of each model's predictions across k = 10 repeated iterations per participant; results are shown in Table 1. For Gemini 1.5 Pro, single‐iteration reliability was consistently excellent across both timepoints. At *T*_0_, ICC(2,1) values ranged from 0.905 (Conscientiousness) to 0.984 (Agreeableness), and at *T*_−1_, from 0.896 (Conscientiousness) to 0.982 (Agreeableness). ICC(2,*k*) values for the averages of 10 iterations were ≥ 0.989 for all traits at both timepoints, indicating near‐perfect reliability when aggregating outputs. In contrast, GPT‐4o exhibited lower stability across repeated iterations. At *T*_0_, ICC(2,1) values ranged from 0.699 (Conscientiousness) to 0.890 (Agreeableness), and at *T*_−1_, from 0.641 (Conscientiousness) to 0.887 (Agreeableness). Nonetheless, ICC(2,*k*) values for averaged predictions remained high (≥ 0.947), highlighting that aggregating multiple iterations substantially improved reliability despite higher variability in individual outputs. Taken together, Gemini 1.5 Pro produced more consistent single‐run personality estimates than GPT‐4o, with both models achieving excellent reliability for aggregated predictions across repetitions.

**TABLE 1:** Intraclass correlation coefficients for single and average iterations of Gemini 1.5 Pro and GPT‐4o across timepoints.

Model	Trait	*T*_0_		*T*_−1_	
		ICC(2,1)	ICC(2,*k*)	ICC(2,1)	ICC(2,*k*)
Gemini 1.5 Pro	Extraversion	0.961	0.996	0.963	0.996
	Agreeableness	0.984	0.998	0.982	0.998
	Conscientiousness	0.905	0.990	0.896	0.989
	Neuroticism	0.913	0.991	0.922	0.992
	Openness	0.959	0.996	0.949	0.995
GPT‐4o	Extraversion	0.842	0.982	0.834	0.980
	Agreeableness	0.890	0.988	0.887	0.987
	Conscientiousness	0.699	0.959	0.641	0.947
	Neuroticism	0.771	0.971	0.754	0.968
	Openness	0.848	0.982	0.806	0.977

*Note:* ICCs were computed using a two‐way random‐effects model (absolute agreement, ICC[2,1] and ICC[2,*k*]). ICC(2,1): reliability of single iterations; ICC(2,*k*): reliability of the average of *k* = 10 iterations.

### 3.2 Stability of LLM Personality Inferences Over Time

To assess the stability of LLM personality inferences over time, we examined the Spearman correlations between inferred traits across two consecutive years of Facebook posting activity (*T*_0_ and *T*_−1_) for both Gemini 1.5 Pro and GPT‐4o, controlling for sex and age. Note that all examined inferences were based on average scores across 10 repeated iterations per participant to ensure greater measurement reliability. Results are reported in Table 2. Gemini 1.5 Pro exhibited moderate temporal stability for Extraversion (*ρ* = 0.47), Openness (*ρ* = 0.53), and Agreeableness (*ρ* = 0.45), with somewhat lower stability for Conscientiousness (*ρ* = 0.38) and Neuroticism (*ρ* = 0.39). GPT‐4o showed similar patterns, with moderate stability for Extraversion (*ρ* = 0.57), Openness (*ρ* = 0.58), and Agreeableness (*ρ* = 0.45), but lower stability for Conscientiousness (*ρ* = 0.37) and Neuroticism (*ρ* = 0.48). Notably, stability was generally highest when personality inferences were averaged across the two models, with Extraversion (*ρ* = 0.57), Agreeableness (*ρ* = 0.51), Conscientiousness (*ρ* = 0.44), and Openness (*ρ* = 0.60) demonstrating improved correlation coefficients. These findings suggest that combining outputs from LLMs tends to enhance the stability of LLM‐derived personality estimates over time.

**TABLE 2:** Stability and convergence of LLM personality inferences (*N* = 1214).

Trait	Stability over time			Cross‐model agreement		
	Gemini 1.5 Pro	GPT‐4o	LLM average	*T*_0_	*T*_−1_	*T*_0_–*T*_−1_ average
Extraversion	0.47	0.57	0.57	0.77	0.77	0.83
Agreeableness	0.45	0.45	0.51	0.69	0.70	0.76
Conscientiousness	0.38	0.37	0.44	0.54	0.59	0.64
Neuroticism	0.39	0.48	0.46	0.53	0.53	0.58
Openness	0.53	0.58	0.60	0.78	0.71	0.79

*Note:* Partial Spearman correlations are presented controlling for sex and age. Correlations are all significant at *p* < 0.001.

### 3.3 Stability of Personality Inferences Across LLM Models

To determine the similarity of personality inferences across different LLMs, we computed partial Spearman correlations between the inferences generated by Gemini 1.5 Pro and GPT‐4o at both *T*_0_ and *T*_−1_, controlling for sex and age. As in stability analyses, the examined inferences were based on average scores across 10 repeated iterations per participant. Results are reported in Table 2. Across both time points, Extraversion and Openness consistently showed the strongest agreement between the two models. At *T*_0_, agreement was particularly high for Extraversion (*ρ* = 0.77) and Openness (*ρ* = 0.78), followed by Agreeableness (*ρ* = 0.69), Neuroticism (*ρ* = 0.53), and Conscientiousness (*ρ* = 0.54). At *T*_−1_, cross‐model correlations were similarly strong, with Extraversion (*ρ* = 0.77), Openness (*ρ* = 0.71), Agreeableness (*ρ* = 0.70), Neuroticism (*ρ* = 0.53), and Conscientiousness (*ρ* = 0.59) all demonstrating robust agreement.

Moreover, cross‐model agreement was further amplified when comparing average personality inferences across both years for each model, with the highest correlations observed for Extraversion (*ρ* = 0.83) and Openness (*ρ* = 0.79), followed by Agreeableness (*ρ* = 0.76), Conscientiousness (*ρ* = 0.64), and Neuroticism (*ρ* = 0.58). These findings indicate that averaging predictions across time points enhances convergence between models, potentially by mitigating transient fluctuations in language use and capturing more stable aspects of personality. Overall, the generally higher correlations observed for cross‐model agreement compared to within‐model temporal stability suggest that individual differences in language use over time contributed more to variations in personality inferences than fundamental differences between the models themselves.

### 3.4 Convergent Validity of LLM Inferences Compared to Self‐Reports

**FIGURE 2:**
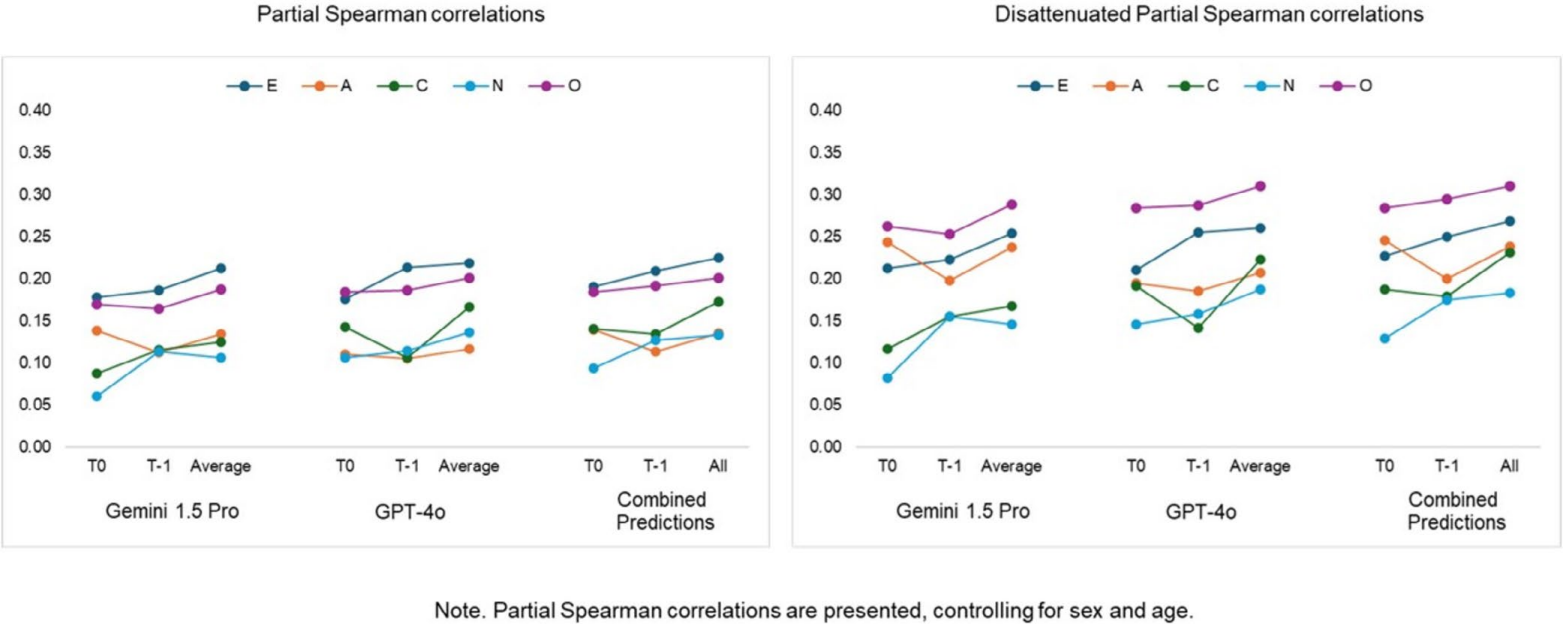
Correlations between TIPI scores and LLM inferences by LLM and prediction combination (*N* = 1214).

The convergent validity of the LLMs in inferring Big Five traits from Facebook posting data was assessed by correlating average LLM inferences across 10 iterations with self‐reported personality traits measured using the TIPI, while controlling for sex and age. Again, the examined inferences were based on average scores across 10 repeated iterations per participant. Results are reported in Figure 2 and Table 3, presented by LLM (Gemini 1.5 Pro and GPT‐4o), year of social media activity (*T*_0_, the year immediately preceding the TIPI assessment, and *T*_−1_, the year before *T*_0_), and combinations of predictions across years and models.

**TABLE 3:** Correlations between TIPI scores and LLM inferences by LLM and prediction combination (*N* = 1214).

	Gemini 1.5 pro			GPT‐4o			Combined models		
Trait	*T*_0_	*T*_−1_	Average	*T*_0_	*T*_−1_	Average	*T*_0_	*T*_−1_	All
Extraversion	0.18 (0.21)	0.19 (0.22)	0.21 (0.25)	0.18 (0.21)	0.21 (0.25)	0.22 (0.26)	0.19 (0.23)	0.21 (0.25)	0.23 (0.27)
Agreeableness	0.14 (0.24)	0.11 (0.20)	0.13 (0.24)	0.11 (0.19)	0.11 (0.19)	0.12 (0.21)	0.14 (0.25)	0.11 (0.20)	0.14 (0.24)
Conscientiousness	0.09* (0.12)	0.12 (0.16)	0.13 (0.17)	0.14 (0.19)	0.11 (0.14)	0.17 (0.22)	0.14 (0.19)	0.13 (0.18)	0.17 (0.23)
Neuroticism	0.06* (0.08)	0.11 (0.16)	0.11 (0.15)	0.11 (0.15)	0.12 (0.16)	0.14 (0.19)	0.09* (0.13)	0.13 (0.17)	0.13 (0.18)
Openness	0.17 (0.26)	0.16 (0.25)	0.19 (0.29)	0.18 (0.28)	0.19 (0.29)	0.20 (0.31)	0.18 (0.28)	0.19 (0.29)	0.20 (0.31)

*Note:* The score for Neuroticism is Emotional Stability reversed. Partial Spearman correlations are reported, controlling for sex and age. Correlations are all significant at *p* < 0.001, except for correlations marked with an asterisk, which are significant at *p* < 0.05. Values in parentheses are disattenuated correlations.

For Gemini 1.5 Pro, correlations at *T*_0_ ranged from *ρ* = 0.06 (disattenuated *ρ* = 0.08, Neuroticism) to *ρ* = 0.18 (disattenuated *ρ* = 0.21, Extraversion), while at *T*_−1_, correlations ranged from *ρ* = 0.11 (disattenuated *ρ* = 0.16, Neuroticism) to *ρ* = 0.19 (disattenuated *ρ* = 0.22, Extraversion). When averaging Gemini inferences across years, Extraversion (*ρ* = 0.21, disattenuated *ρ* = 0.25) and Openness (*ρ* = 0.19, disattenuated *ρ* = 0.29) showed the strongest associations, with Agreeableness (*ρ* = 0.13, disattenuated *ρ* = 0.24), Conscientiousness (*ρ* = 0.13, disattenuated *ρ* = 0.17), and Neuroticism (*ρ* = 0.11, disattenuated *ρ* = 0.15) showing weaker but significant correlations.

For GPT‐4o, correlations at *T*_0_ ranged from *ρ* = 0.11 (disattenuated *ρ* = 0.15, Neuroticism) to *ρ* = 0.18 (disattenuated *ρ* = 0.28, Openness), while at *T*_−1_, they ranged from *ρ* = 0.11 (disattenuated *ρ* = 0.14, Conscientiousness) to *ρ* = 0.21 (disattenuated *ρ* = 0.25, Extraversion). Averaging across years, Extraversion (*ρ* = 0.22, disattenuated *ρ* = 0.26) and Openness (*ρ* = 0.20, disattenuated *ρ* = 0.31) again showed the strongest validity, while Agreeableness (*ρ* = 0.12, disattenuated *ρ* = 0.21), Conscientiousness (*ρ* = 0.17, disattenuated *ρ* = 0.22), and Neuroticism (*ρ* = 0.14, disattenuated *ρ* = 0.19) demonstrated smaller but significant correlations.

Combining inferences from both LLMs generally improved convergent validity. At *T*_0_, combined correlations ranged from *ρ* = 0.09 (disattenuated *ρ* = 0.13, Neuroticism) to *ρ* = 0.19 (disattenuated *ρ* = 0.23, Extraversion), while at *T*_−1_, they ranged from *ρ* = 0.11 (disattenuated *ρ* = 0.20, Agreeableness) to *ρ* = 0.21 (disattenuated *ρ* = 0.25, Extraversion). When combining inferences across all possible model and timepoint averages, Extraversion (*ρ* = 0.23, disattenuated *ρ* = 0.27) and Openness (*ρ* = 0.20, disattenuated *ρ* = 0.31) showed the strongest associations, with Agreeableness (*ρ* = 0.14, disattenuated *ρ* = 0.24), Conscientiousness (*ρ* = 0.17, disattenuated *ρ* = 0.23), and Neuroticism (*ρ* = 0.13, disattenuated *ρ* = 0.18) also demonstrating significant, albeit more modest, correlations. These findings indicate that, although effect sizes were generally small, both Gemini 1.5 Pro and GPT‐4o captured meaningful variance in self‐reported personality traits, with Extraversion and Openness consistently showing the strongest associations.

### 3.5 Accuracy of LLM Inferences Compared to a Random Baseline Model

Finally, we evaluated the predictive accuracy of the LLM‐derived personality inferences by comparing their error metrics (MAE, MSE, RMSE) to those from a baseline model predicting traits with random guesses. Additionally, we examined predictive *R* ^2^, representing the proportion of variance in self‐reported scores explained by the model relative to random guesses, where values above zero indicate better‐than‐chance predictions. Here we only comment on model and data combinations that showed the best accuracy compared to random guesses. Full results for all models and traits are reported in Table S1. For all traits except Extraversion, combining predictions across LLMs and timepoints consistently produced the most accurate results. For Agreeableness, the combined average achieved MAE = 1.36, MSE = 2.64, RMSE = 1.62, and average predictive *R* ^2^ = 0.55, compared to higher errors from the random baseline (MAE = 1.98; MSE = 5.85; RMSE = 2.42). Conscientiousness averaged predictions were only slightly better than chance (MAE = 2.01, MSE = 5.22, RMSE = 2.28, *R* ^2^ = 0.17; Random baseline: MAE = 2.05, MSE = 6.26, RMSE = 2.50). Neuroticism showed clearer improvements (MAE = 1.20, MSE = 2.17, RMSE = 1.47, *R* ^2^ = 0.57; Random baseline: MAE = 1.85, MSE = 5.09, RMSE = 2.26), and Openness achieved the highest accuracy overall (MAE = 0.98, MSE = 1.52, RMSE = 1.23, *R* ^2^ = 0.72; Random baseline: MAE = 1.90, MSE = 5.39, RMSE = 2.32). In contrast with the other traits, Extraversion showed the best results from GPT‐4o predictions averaged across timepoints (MAE = 1.45, MSE = 3.09, RMSE = 1.76, *R* ^2^ = 0.45; Random baseline: MAE = 1.93, MSE = 5.61, RMSE = 2.37). These findings demonstrate that integrating outputs either from both LLMs or across measurement occasions substantially improved predictive accuracy over random guessing, particularly for Openness, Agreeableness, and Neuroticism.

## 4 Discussion

This study investigated the ability of large language models (LLMs) to infer personality traits from Facebook activity, focusing on the stability of these inferences over time and their convergence across different models, and how closely they align with self‐reports. We examined the performance of two state‐of‐the‐art LLMs, Gemini 1.5 Pro and GPT‐4o, in predicting self‐reported Big Five personality traits based on users' Facebook posting activity spanning two consecutive years. First, we comment on results for convergence with self‐reports. Overall, correlations between LLM‐derived inferences and TIPI scores were small, and at their highest when averaging inferences across years and LLMs, with correlations with TIPI scores ranging from 0.18 (Neuroticism) to 0.31 (Openness) after correcting them from measurement error of self‐reports. The strongest associations were found for Openness and Extraversion, aligning with previous research that identified these traits as more readily predictable from digital footprints (Azucar et al. 2018; Park et al. 2015). Combining inferences across both years and models generally yielded slight improvements in correlations with self‐reported traits, suggesting that aggregating predictions can enhance validity. However, the overall modest correlations highlight the limitations of relying solely on social media data for accurate personality assessment. These findings align with recent work by Peters and Matz (2024), who also found small correlations between LLM inferences and self‐reported Big Five traits.

Of note, when comparing the central tendency of LLM inferences to self‐reports, the sample mean of Neuroticism and especially Openness was inferred with reasonable accuracy by both examined LLMs (i.e., Gemini 1.5 Pro and GPT‐4o). In contrast, we observed a systematic underestimation of Agreeableness and Conscientiousness, and a slight overestimation of Extraversion. This emerging pattern is in line with findings previously reported by Peters and Matz (2024) using GPT‐4, and suggests potential biases in how LLMs interpret language cues related to these traits or reflects peculiarities of personal expression on Facebook, biases that persisted across both models and timepoints examined here. The mismatch between the verbal labels used in the LLM prompts and those of the original TIPI response could also partially explain the systematic under‐ or overestimation observed for certain traits and should be addressed in future studies to improve comparability.

Interestingly, Gemini 1.5 Prodemonstrated lower variance across repetitions compared to GPT‐4o, indicating greater stability in predictions. Our reliability analyses further showed Gemini 1.5 Pro inferences were highly stable even in single iterations, while GPT‐4o's outputs varied considerably over multiple repetitions. In this context, averaging predictions by the same LLM across multiple iterations may prove an effective strategy for both models, supporting repeated prompting as a best practice when using LLMs for personality inference (e.g., Bodroža et al. 2024; Cao and Kosinski 2024; Peters and Matz 2024).

Our study also investigated the temporal stability and convergence across models of LLM inferences. Our results also indicate that LLM‐derived personality inferences exhibit varying degrees of stability across time. Inferred Extraversion and Openness showed the highest stability, with moderate‐to‐strong correlations between inferences derived from two consecutive years of Facebook activity for both Gemini 1.5 Pro and GPT‐4o. This suggests that these traits may be more consistently expressed and detected in digital footprints over time. Agreeableness, Conscientiousness, and Neuroticism exhibited the lowest stability across both models, suggesting that these traits might be more challenging to infer consistently from the type of digital footprints analyzed in this study. Again, these findings are consistent with previous studies using machine learning models that showed the highest accuracy in predicting Extraversion and Openness compared to the other Big Five traits when leveraging social media data (e.g., Azucar et al. 2018).

A key finding of this study is also the moderate‐to‐strong agreement between different LLMs (i.e., Gemini 1.5 Pro and GPT‐4o) in their personality inferences. Correlations between the models' predictions were even higher year‐to‐year stability within each model, particularly for Extraversion and Openness. This convergence suggests that despite differences in their underlying architectures and training data, both LLMs are capturing similar signals from the Facebook data that are relevant to personality. The even higher cross‐model agreement observed when averaging predictions across both years further supports this notion, indicating that combining inferences across time can mitigate the impact of transient fluctuations in language use and reveal more stable underlying characteristics.

Beyond practical implications for personality assessment, these findings carry important theoretical implications. They provide novel, data‐driven evidence supporting trait theories' core proposition that personality traits tend to be relatively stable dispositions persisting across time and contexts. The moderate consistency of LLM‐inferred traits across 2 years suggests that linguistic signals of personality endure, reinforcing the Big Five framework as a model of enduring tendencies (Roberts et al. 2006). Unlike earlier computational studies limited to a single snapshot of social media language (see Azucar et al. 2018 for a meta‐analysis), our approach demonstrates that personality expression in natural language remains detectable across extended periods.

Crucially, our results extend classic theories of personality expression (e.g., Yarkoni 2010) by empirically demonstrating which traits “leak” most reliably into digital language. Extraversion and Openness not only showed the highest temporal stability but also the strongest cross‐model agreement, aligning with prior research identifying these traits as more externally visible and easier to infer from “thin slices” of behavior (Vazire 2010). This pattern is consistent with the *self–other knowledge asymmetry* model, which posits that outsiders, here, the LLMs, are better at judging traits that are highly observable, lending credibility to our computational inferences as reflections of genuine trait expression.

Furthermore, the moderate agreement between LLM‐inferred and self‐reported traits, especially for Extraversion and Openness, evident both in correlations and in part in comparable mean scores, supports the convergent validity of LLM‐based assessments. It indicates that computational methods and traditional self‐reports tap overlapping aspects of underlying personality constructs. Collectively, these findings extend trait theory by showing that stable, trait‐consistent patterns are evident in everyday language over time, and that computationally derived inferences align to a moderate degree with established self‐report measures, highlighting the enduring nature of personality as it manifests in digital behavior.

### 4.1 Limitations and Future Directions

This study has several limitations. First, the sample consisted mainly of young, well‐educated Italian Facebook users, limiting the generalizability of our findings. Second, our data were collected in early 2018, and since user posts were retrieved retroactively via the Facebook API, they included status updates dating back to as early as 2016. Given that online language use has likely evolved since then, we anticipate that LLMs trained on more recent data could perform even better at inferring personality from contemporary text. Nonetheless, it is worth noting that our data are comparably more recent than those analyzed by Peters and Matz (2024), which were collected using the MyPersonality app from 2007 to 2012. The reliance on older data, common in this research area, underscores the urgent need for more contemporary datasets and increased data openness from social media platforms, as advocated by Montag et al. (2021).

Third, as noted earlier, our reliance on the TIPI to measure ground truth personality, while practical for its brevity, may have introduced measurement error due to the low number of items per trait. Although we provided disattenuated correlations to correct for this, future studies could use more comprehensive personality inventories to enhance reliability. Fourth, although our prompts matched the TIPI's 7‐point numerical scale, the verbal labels used did not correspond exactly to those of the TIPI, potentially introducing discrepancies between self‐reported and LLM‐inferred scores. Future research should ensure closer alignment of scale anchors to improve comparability.

Fifth, the study focused solely on Facebook data, capturing only one facet of individuals' online behavior. Future work should integrate data from multiple platforms to provide a more holistic picture of digital footprints. Sixth, we used two widely available proprietary LLMs (Gemini 1.5 Pro and GPT‐4o) to assess predictive accuracy in models easily accessible to researchers, professionals, and the general public. However, using these closed‐source models limits transparency due to their proprietary architectures. Future research should explore open‐weight or source‐available models, such as Meta's LLaMA or fully open‐source alternatives like Mistral, to allow for greater transparency, replicability, and control over model parameters. Incorporating these models could provide deeper insights into how specific architectures and training data influence personality inferences, enabling the research community to systematically evaluate and refine prompting strategies for unobtrusive personality assessment.

Finally, although the privacy rights of participants were always carefully considered, submitting user posts to proprietary LLM APIs carries inherent privacy limitations. However, OpenAI and Google state that prompts and responses are not used for model training and are retained only temporarily for abuse monitoring (up to 30 days for OpenAI and 55 days for Google), which helps mitigate potential risks. Nevertheless, future research could benefit from exploring locally hosted or open‐weight models to further strengthen privacy protections.

## 5 Conclusion

Despite the aforementioned limitations, this study provides valuable insights into the capabilities and limitations of LLMs for longitudinal personality inference. Our findings contribute to the growing literature on computational social science and the use of LLMs for psychological profiling, building upon previous work that primarily focused on static snapshots of online behavior (Azucar et al. 2018; Kosinski et al. 2013; Park et al. 2015). The findings highlight the importance of considering temporal dynamics and cross‐model convergence when evaluating the accuracy and validity of LLM‐based psychological assessments. Future research should explore the factors that contribute to the stability and instability of personality inferences over time, investigate methods for improving the accuracy of LLMs in predicting traits, and further examine the ethical implications of using these models for personality profiling (Kosinski 2024; Matz et al. 2020). As LLMs continue to evolve, understanding their strengths and weaknesses in the domain of psychological assessment will be crucial for their responsible and effective application in research and industry.

## Author Contributions

**Davide Marengo:** conceptualization, data curation, formal analysis, investigation, methodology, project administration, software, visualization, writing – original draft, writing – review and editing; **Christian Montag:** methodology, validation, writing – original draft, writing – review and editing; **Michele Settanni:** conceptualization, investigation, methodology, project administration, supervision, validation, writing – original draft.

## Conflicts of Interest

The authors declare no conflicts of interest.

## Supporting information


**Data S1:** jopy70019‐sup‐0001‐Supinfo1.zip.
