# Supplementary material for: Inferring Personality From Social Media Activity Using Large Language Models: Cross‐Model Agreement, Temporal Stability, and Convergent Validity With Self‐Reports
Source: J Pers. 2025 Sep 2;94(4):525–34. doi: 10.1111/jopy.70019 (PMC13359307; doi:10.1111/jopy.70019)
Supplement: Supplementary file 1 — Data S1: jopy70019‐sup‐0001‐Supinfo1.zip. [file JOPY-94-525-s001.zip › jopy70019-sup-0001-Supinfo1/jopy70019-sup-0001-Supinfo1.docx]

**Supplementary material**

**Prompt**

*Recall the personality traits: extraversion, agreeableness, conscientiousness, neuroticism, and openness to experience.*

- *Individuals with a high level of extraversion may see themselves as someone who is talkative, sociable, energetic, or enthusiastic. Individuals with a low level of extraversion may see themselves as someone who is reserved, quiet, or inhibited.*
- *Individuals with a high level of agreeableness may see themselves as someone who is helpful, kind, forgiving, or sympathetic. Individuals with a low level of agreeableness may see themselves as someone who is critical, demanding, stubborn, or rude.*
- *Individuals with a high level of conscientiousness may see themselves as someone who is efficient, organized, reliable, or thorough. Individuals with a low level of conscientiousness may see themselves as someone who is careless, disorganized, impulsive, or lazy.*
- *Individuals with a high level of neuroticism may see themselves as someone who is sensitive, tense, moody, or easily upset. Individuals with a low level of neuroticism may see themselves as someone who is secure, calm, relaxed, or emotionally stable.*
- *Individuals with a high level of openness to experience may see themselves as someone who is curious, imaginative, artistic, or unconventional. Individuals with a low level of openness to experience may see themselves as someone who is conventional, prefers routine, or has limited interests.*

*Consider the following Facebook data by the same person, including timestamps of activity, status and story updates, and received Likes:*

*USER FACEBOOK DATA INSERTED HERE*

*For each trait (extraversion, agreeableness, conscientiousness, neuroticism, openness), rate the person using the following label:*

*1 (very low)*

*2 (low)*

*3 (low to moderate)*

*4 (moderate)*

*5 (moderate to high)*

*6 (high)*

*7 (very high).*

*Provide the scores using a comma-separated response in the format: score for extraversion, score for agreeableness, score for conscientiousness, score for neuroticism, score for openness. Do not give an explanation.*
