# Supplementary material for: Inferring Personality From Social Media Activity Using Large Language Models: Cross‐Model Agreement, Temporal Stability, and Convergent Validity With Self‐Reports
Source: J Pers. 2025 Sep 2;94(4):525–34. doi: 10.1111/jopy.70019 (PMC13359307; doi:10.1111/jopy.70019)
Supplement: Supplementary file 1 — Data S1: jopy70019‐sup‐0001‐Supinfo1.zip. [file JOPY-94-525-s001.zip › jopy70019-sup-0001-Supinfo1/jopy70019-sup-0005-TableS1.docx]

Table S1. Accuracy of LLM inferences Compared to a Random Baseline Model by Trait and LLM combination

| Trait | Model | MAE | MSE | RMSE | Predictive_R2_mean | Predictive_R2_min | Predictive_R2_max | Random_MAE_mean | Random_MAE_min | Random_MAE_max | Random_MSE_mean | Random_MSE_min | Random_MSE_max | Random_RMSE_mean | Random_RMSE_min | Random_RMSE_max |
| --- | --- | --- | --- | --- | --- | --- | --- | --- | --- | --- | --- | --- | --- | --- | --- | --- |
| Agreeableness | Combined Models (T_0_) | 1.40 | 2.85 | 1.69 | 0.51 | 0.55 | 0.49 | 1.98 | 1.92 | 2.09 | 5.85 | 5.53 | 6.37 | 2.42 | 2.35 | 2.52 |
| Agreeableness | Combined Models (T_0_-T_-1_) | 1.36 | 2.64 | 1.62 | 0.55 | 0.59 | 0.52 | 1.98 | 1.92 | 2.09 | 5.85 | 5.53 | 6.37 | 2.42 | 2.35 | 2.52 |
| Agreeableness | Combined Models (T_1_) | 1.37 | 2.71 | 1.65 | 0.54 | 0.58 | 0.51 | 1.98 | 1.92 | 2.09 | 5.85 | 5.53 | 6.37 | 2.42 | 2.35 | 2.52 |
| Agreeableness | Gemini 1.5 pro (T_0_) | 1.40 | 2.89 | 1.70 | 0.51 | 0.55 | 0.48 | 1.98 | 1.92 | 2.09 | 5.85 | 5.53 | 6.37 | 2.42 | 2.35 | 2.52 |
| Agreeableness | Gemini 1.5 pro (T_0_-T_-1_) | 1.36 | 2.68 | 1.64 | 0.54 | 0.58 | 0.52 | 1.98 | 1.92 | 2.09 | 5.85 | 5.53 | 6.37 | 2.42 | 2.35 | 2.52 |
| Agreeableness | Gemini 1.5 pro (T_-1_) | 1.40 | 2.87 | 1.69 | 0.51 | 0.55 | 0.48 | 1.98 | 1.92 | 2.09 | 5.85 | 5.53 | 6.37 | 2.42 | 2.35 | 2.52 |
| Agreeableness | GPT4o (T_0_) | 1.45 | 3.03 | 1.74 | 0.48 | 0.52 | 0.45 | 1.98 | 1.92 | 2.09 | 5.85 | 5.53 | 6.37 | 2.42 | 2.35 | 2.52 |
| Agreeableness | GPT4o (T_0_-T_-1_) | 1.38 | 2.73 | 1.65 | 0.53 | 0.57 | 0.51 | 1.98 | 1.92 | 2.09 | 5.85 | 5.53 | 6.37 | 2.42 | 2.35 | 2.52 |
| Agreeableness | GPT4o (T_-1_) | 1.38 | 2.76 | 1.66 | 0.53 | 0.57 | 0.50 | 1.98 | 1.92 | 2.09 | 5.85 | 5.53 | 6.37 | 2.42 | 2.35 | 2.52 |
| Conscientiousness | Combined Models (T_0_) | 2.01 | 5.26 | 2.29 | 0.16 | 0.20 | 0.13 | 2.05 | 2.01 | 2.11 | 6.26 | 6.06 | 6.56 | 2.50 | 2.46 | 2.56 |
| Conscientiousness | Combined Models (T_0_-T_-1_) | 2.01 | 5.22 | 2.28 | 0.17 | 0.20 | 0.14 | 2.05 | 2.01 | 2.11 | 6.26 | 6.06 | 6.56 | 2.50 | 2.46 | 2.56 |
| Conscientiousness | Combined Models (T_1_) | 2.02 | 5.29 | 2.30 | 0.16 | 0.19 | 0.13 | 2.05 | 2.01 | 2.11 | 6.26 | 6.06 | 6.56 | 2.50 | 2.46 | 2.56 |
| Conscientiousness | Gemini 1.5 pro (T_0_) | 2.01 | 5.36 | 2.32 | 0.14 | 0.18 | 0.12 | 2.05 | 2.01 | 2.11 | 6.26 | 6.06 | 6.56 | 2.50 | 2.46 | 2.56 |
| Conscientiousness | Gemini 1.5 pro (T_0_-T_-1_) | 2.00 | 5.26 | 2.29 | 0.16 | 0.20 | 0.13 | 2.05 | 2.01 | 2.11 | 6.26 | 6.06 | 6.56 | 2.50 | 2.46 | 2.56 |
| Conscientiousness | Gemini 1.5 pro (T_-1_) | 2.01 | 5.32 | 2.31 | 0.15 | 0.19 | 0.12 | 2.05 | 2.01 | 2.11 | 6.26 | 6.06 | 6.56 | 2.50 | 2.46 | 2.56 |
| Conscientiousness | GPT4o (T_0_) | 2.02 | 5.29 | 2.30 | 0.16 | 0.19 | 0.13 | 2.05 | 2.01 | 2.11 | 6.26 | 6.06 | 6.56 | 2.50 | 2.46 | 2.56 |
| Conscientiousness | GPT4o (T_0_-T_-1_) | 2.01 | 5.25 | 2.29 | 0.16 | 0.20 | 0.13 | 2.05 | 2.01 | 2.11 | 6.26 | 6.06 | 6.56 | 2.50 | 2.46 | 2.56 |
| Conscientiousness | GPT4o (T_-1_) | 2.02 | 5.35 | 2.31 | 0.15 | 0.18 | 0.12 | 2.05 | 2.01 | 2.11 | 6.26 | 6.06 | 6.56 | 2.50 | 2.46 | 2.56 |
| Extraversion | Combined Models (T_0_) | 1.50 | 3.35 | 1.83 | 0.40 | 0.42 | 0.36 | 1.93 | 1.85 | 1.97 | 5.61 | 5.24 | 5.77 | 2.37 | 2.29 | 2.40 |
| Extraversion | Combined Models (T_0_-T_-1_) | 1.52 | 3.34 | 1.83 | 0.41 | 0.42 | 0.36 | 1.93 | 1.85 | 1.97 | 5.61 | 5.24 | 5.77 | 2.37 | 2.29 | 2.40 |
| Extraversion | Combined Models (T_1_) | 1.60 | 3.76 | 1.94 | 0.33 | 0.35 | 0.28 | 1.93 | 1.85 | 1.97 | 5.61 | 5.24 | 5.77 | 2.37 | 2.29 | 2.40 |
| Extraversion | Gemini 1.5 pro (T_0_) | 1.59 | 3.81 | 1.95 | 0.32 | 0.34 | 0.27 | 1.93 | 1.85 | 1.97 | 5.61 | 5.24 | 5.77 | 2.37 | 2.29 | 2.40 |
| Extraversion | Gemini 1.5 pro (T_0_-T_-1_) | 1.60 | 3.81 | 1.95 | 0.32 | 0.34 | 0.27 | 1.93 | 1.85 | 1.97 | 5.61 | 5.24 | 5.77 | 2.37 | 2.29 | 2.40 |
| Extraversion | Gemini 1.5 pro (T_-1_) | 1.72 | 4.39 | 2.10 | 0.22 | 0.24 | 0.16 | 1.93 | 1.85 | 1.97 | 5.61 | 5.24 | 5.77 | 2.37 | 2.29 | 2.40 |
| Extraversion | GPT4o (T_0_) | 1.47 | 3.21 | 1.79 | 0.43 | 0.44 | 0.39 | 1.93 | 1.85 | 1.97 | 5.61 | 5.24 | 5.77 | 2.37 | 2.29 | 2.40 |
| Extraversion | GPT4o (T_0_-T_-1_) | 1.45 | 3.09 | 1.76 | 0.45 | 0.47 | 0.41 | 1.93 | 1.85 | 1.97 | 5.61 | 5.24 | 5.77 | 2.37 | 2.29 | 2.40 |
| Extraversion | GPT4o (T_-1_) | 1.52 | 3.42 | 1.85 | 0.39 | 0.41 | 0.35 | 1.93 | 1.85 | 1.97 | 5.61 | 5.24 | 5.77 | 2.37 | 2.29 | 2.40 |
| Neuroticism | Combined Models (T_0_) | 1.24 | 2.30 | 1.52 | 0.55 | 0.56 | 0.53 | 1.85 | 1.80 | 1.90 | 5.09 | 4.85 | 5.28 | 2.26 | 2.20 | 2.30 |
| Neuroticism | Combined Models (T_0_-T_-1_) | 1.20 | 2.17 | 1.47 | 0.57 | 0.59 | 0.55 | 1.85 | 1.80 | 1.90 | 5.09 | 4.85 | 5.28 | 2.26 | 2.20 | 2.30 |
| Neuroticism | Combined Models (T_1_) | 1.22 | 2.24 | 1.50 | 0.56 | 0.58 | 0.54 | 1.85 | 1.80 | 1.90 | 5.09 | 4.85 | 5.28 | 2.26 | 2.20 | 2.30 |
| Neuroticism | Gemini 1.5 pro (T_0_) | 1.28 | 2.44 | 1.56 | 0.52 | 0.54 | 0.50 | 1.85 | 1.80 | 1.90 | 5.09 | 4.85 | 5.28 | 2.26 | 2.20 | 2.30 |
| Neuroticism | Gemini 1.5 pro (T_0_-T_-1_) | 1.23 | 2.27 | 1.51 | 0.55 | 0.57 | 0.53 | 1.85 | 1.80 | 1.90 | 5.09 | 4.85 | 5.28 | 2.26 | 2.20 | 2.30 |
| Neuroticism | Gemini 1.5 pro (T_-1_) | 1.26 | 2.40 | 1.55 | 0.53 | 0.55 | 0.51 | 1.85 | 1.80 | 1.90 | 5.09 | 4.85 | 5.28 | 2.26 | 2.20 | 2.30 |
| Neuroticism | GPT4o (T_0_) | 1.25 | 2.37 | 1.54 | 0.53 | 0.55 | 0.51 | 1.85 | 1.80 | 1.90 | 5.09 | 4.85 | 5.28 | 2.26 | 2.20 | 2.30 |
| Neuroticism | GPT4o (T_0_-T_-1_) | 1.21 | 2.20 | 1.48 | 0.57 | 0.58 | 0.55 | 1.85 | 1.80 | 1.90 | 5.09 | 4.85 | 5.28 | 2.26 | 2.20 | 2.30 |
| Neuroticism | GPT4o (T_-1_) | 1.24 | 2.30 | 1.52 | 0.55 | 0.56 | 0.53 | 1.85 | 1.80 | 1.90 | 5.09 | 4.85 | 5.28 | 2.26 | 2.20 | 2.30 |
| Openness | Combined Models (T_0_) | 1.04 | 1.72 | 1.31 | 0.68 | 0.69 | 0.67 | 1.90 | 1.86 | 1.96 | 5.39 | 5.24 | 5.62 | 2.32 | 2.29 | 2.37 |
| Openness | Combined Models (T_0_-T_-1_) | 0.98 | 1.52 | 1.23 | 0.72 | 0.73 | 0.71 | 1.90 | 1.86 | 1.96 | 5.39 | 5.24 | 5.62 | 2.32 | 2.29 | 2.37 |
| Openness | Combined Models (T_1_) | 0.99 | 1.60 | 1.26 | 0.70 | 0.72 | 0.70 | 1.90 | 1.86 | 1.96 | 5.39 | 5.24 | 5.62 | 2.32 | 2.29 | 2.37 |
| Openness | Gemini 1.5 pro (T_0_) | 1.07 | 1.80 | 1.34 | 0.67 | 0.68 | 0.66 | 1.90 | 1.86 | 1.96 | 5.39 | 5.24 | 5.62 | 2.32 | 2.29 | 2.37 |
| Openness | Gemini 1.5 pro (T_0_-T_-1_) | 1.00 | 1.59 | 1.26 | 0.71 | 0.72 | 0.70 | 1.90 | 1.86 | 1.96 | 5.39 | 5.24 | 5.62 | 2.32 | 2.29 | 2.37 |
| Openness | Gemini 1.5 pro (T_-1_) | 1.04 | 1.72 | 1.31 | 0.68 | 0.69 | 0.67 | 1.90 | 1.86 | 1.96 | 5.39 | 5.24 | 5.62 | 2.32 | 2.29 | 2.37 |
| Openness | GPT4o (T_0_) | 1.06 | 1.83 | 1.35 | 0.66 | 0.67 | 0.65 | 1.90 | 1.86 | 1.96 | 5.39 | 5.24 | 5.62 | 2.32 | 2.29 | 2.37 |
| Openness | GPT4o (T_0_-T_-1_) | 0.99 | 1.58 | 1.26 | 0.71 | 0.72 | 0.70 | 1.90 | 1.86 | 1.96 | 5.39 | 5.24 | 5.62 | 2.32 | 2.29 | 2.37 |
| Openness | GPT4o (T_-1_) | 1.01 | 1.67 | 1.29 | 0.69 | 0.70 | 0.68 | 1.90 | 1.86 | 1.96 | 5.39 | 5.24 | 5.62 | 2.32 | 2.29 | 2.37 |
| **Note.** *MAE*: Mean Absolute Error between the model’s predicted trait scores and self-reported scores; *MSE*: Mean Squared Error between the model’s predicted trait scores and self-reported scores; *RMSE*: Root Mean Squared Error between the model’s predicted trait scores and self-reported scores; ; *Predictive_R2_mean*: Predictive R² computed using the mean random baseline MSE; *Predictive_R2_min*: Predictive R² using the worst-case random baseline (highest random MSE), giving a conservative estimate of model performance; *Predictive_R2_max*: Predictive R² using the best-case random baseline (lowest random MSE); *Random_MAE_mean*: Mean MAE across the 10 random guess repetitions for the given trait; *Random_MAE_min*: Lowest (best-case) MAE among the 10 random guess repetitions; *Random_MAE_max*: Highest (worst-case) MAE among the 10 random guess repetitions; *Random_MSE_mean*: Mean MSE across the 10 random guess repetitions; *Random_MSE_min*: Lowest (best-case) MSE among the 10 random guess repetitions; *Random_MSE_max*: Highest (worst-case) MSE among the 10 random guess repetitions; *Random_RMSE_mean*: Mean RMSE across the 10 random guess repetitions; Random_RMSE_min: Lowest (best-case) RMSE among the 10 random guess repetitions; *Random_RMSE_max*: Highest (worst-case) RMSE among the 10 random guess repetitions. | | | | | | | | | | | | | | | | |
